# The Separation of the National Associations

**Published:** 1896-12

**Authors:** 


### THE SEPARATION OF THE NATIONAL ASSOCIATIONS.

The *Dental Practitioner and Advertiser* probably voices the opinion of some members of the National Association of Dental Faculties when it advocates, in the October number, "that the educational bodies should convene at a time different from that of the meeting of the American Dental Association." Indeed, it has gone so far that the chairman of the Executive Committee has deemed it of sufficient importance to send out a circular letter to the members of that Association to secure an opinion for or against a change of time of meeting. The reasons why such a change should be made are given in the editorial of our contemporary, as follows: "The sessions of the educational bodies greatly detract from the interest of the national professional meeting. Some of the best men in dentistry are members of both. The National Association of Dental Faculties meets and must meet first. The members are tired out when the American assembles, . . . their non-attendance is noted, and they are decidedly missed. . . . It is, then, undoubtedly for the interest of the American Dental Association that it should be relieved of the harassing and perplexing presence of the educational associations."

The main facts as here stated cannot be controverted, but is the remedy suggested the only or the proper one?

To call the meeting of the American Dental Association at one period in the year and the educational bodies at another and in an entirely different place means, in the opinion of the writer, practical death to one or both. If the National Association of Dental Faculties decided to change its time of meeting, the delegates from colleges would be obliged to attend. All these have long distances to travel, and many long journeys involving heavy expense and physical exhaustion. If, then, the separation proposed should be finally decided upon, what would, in all probability, be the result? The men comprising the membership of the National Association of Dental Faculties would refuse to take two long journeys in one year, and, necessity compelling attendance at the "Faculties," they would naturally neglect the other.

Now, these men connected with colleges are, in the main, the life of the American. Without their aid it is questionable whether this Association could be maintained as a national body. This, it is believed, is admitted by all conversant with its affairs.

Aside from this, the inspiriting presence of several related organizations is a decided benefit. The only meeting of the National Association of Dental Faculties that ever convened independently was the one at Chicago, and it was the least valuable, from any point of view, that has ever been held by that organization.

There should be no change in the arrangement of meetings, except in one particular. The next meeting of the American Dental Association, at Old Point Comfort, will be a very important one, and should have a full attendance, free from other entanglements. The suggestion we would make would be for the Executive Committee, or the officers having authority, to issue a call for the meeting of the educational bodies one or two days earlier than usual. The

American does not meet until Tuesday, and it has been customary to call these associations together on the previous Saturday. This allows but two days for the completion of their work. If these bodies were called to meet on Thursday it would give them ample time to finish by Saturday. This would give the members two days of rest before the work of the National Association began,—a time sufficient, it may be supposed, for all practical purposes of recuperation.

It is very questionable whether the Executive Committee of the National Association of Dental Faculties would not exceed its authority were it to call, this year, a midwinter meeting. If any change be made it must first be adopted at the regular annual meeting, and we protest in advance against any such arrangement being settled, by correspondence, as a violation of the policy and methods governing that organization. It is to be hoped that there will be a decided objection to any such irregular work.

